# Effectiveness of Traditional Chinese Medicine Compound JieDuTongLuoShengJin Granules Treatment in Primary Sjögren's Syndrome: A Randomized, Double-Blind, Placebo-Controlled Clinical Trial

**DOI:** 10.1155/2017/1315432

**Published:** 2017-07-10

**Authors:** Ben Li, Jiaqi Hou, Yue Yang, Xuemei Piao, Yueying Chen, Luan Xue, Dan Wang, Jiandong Hu, Guoling Li, Xiangxiang Wu, Yu Sun, Runa Jin

**Affiliations:** ^1^Department of Rheumatology and Immunology, Yueyang Hospital of Integrated Traditional Chinese and Western Medicine, Shanghai University of Traditional Chinese Medicine, Shanghai 200437, China; ^2^Shanghai University of Traditional Chinese Medicine, Shanghai 200437, China; ^3^Department of Ophthalmology, Yueyang Hospital of Integrated Traditional Chinese and Western Medicine, Shanghai University of Traditional Chinese Medicine, Shanghai 200437, China

## Abstract

**Objective:**

To evaluate the clinical therapeutic efficacy and safety of JieDuTongLuoShengJin granules + HCQ in patients with pSS.

**Methods:**

40 patients with low-activity-level pSS and without visceral involvement participated in this study and were randomized to receive either JieDuTongLuoShengJin granules with HCQ or placebo with HCQ. Patients and investigators were blinded to treatment allocation. The primary endpoint was week 12 ESSPRI score, while secondary endpoints included ESSDAI, salivary and lacrimal gland function, and some laboratory variables. Safety-related data were also assessed.

**Results:**

Comparing with the placebo group, the treatment group experienced statistically significant improvement in the mean change from baseline for the primary endpoint of ESSPRI score and also in PGA. Moreover, in comparison with baseline values, the treatment group had significantly improved ESSDAI score, unstimulated saliva flow rate, and several laboratory variables. However, upon comparison of the two groups, there were no significant differences for them. The incidence of AEs was 10.0%, one in treatment group and three in placebo group.

**Conclusion:**

Treatment with a combination of JieDuTongLuoShengJin granules with HCQ is effective in improving patients' subjective symptoms and some objective indicators of pSS. These results indicate that JieDuTongLuoShengJin is promising as a safe and effective treatment of pSS.

## 1. Introduction

Sjögren's syndrome is a systemic diffuse connective tissue disease that is characterized by chronic, autoimmune inflammation of exocrine glands, especially the salivary and lacrimal glands, resulting in glandular hypofunction. In addition to xerostomia and keratoconjunctivitis sicca, Sjögren's syndrome may frequently be accompanied by arthralgia and myalgia and 85% of patients experience severe fatigue. Furthermore, some patients have other serious extraglandular involvement—such as the kidneys, liver, and lungs—and manifest corresponding symptoms [1]. Laboratory manifestations, such as the presence of anti-SSA and anti-SSB autoantibodies and increased serum levels of rheumatoid factor (RF), are due to B cell hyperactivity.

Primary Sjögren's syndrome (pSS) has developed into one of the most common autoimmune rheumatic diseases in the USA, affecting three million people [2]. The prevalence in China is about 0.3% to 0.4%, second only to that of rheumatoid arthritis (RA) [3]. The incidence of Sjögren's syndrome in the elderly is estimated to be 3%-4% [4].

Sjögren's syndrome can have significant impacts on patients' health-related quality of life, leading to many social problems. However, to date there is no curative systemic treatment for pSS. The main goal of treatment is to relieve clinical symptoms, prevent further organ damage due to disease progression, and prolong patients' survival. In recent years, there has been extensive study of new treatment methods for Sjögren's syndrome, including rituximab, belimumab, and others. However, most have failed to show significant improvement in patients' important symptoms such as dryness, fatigue, and pain [5–7]. For those Sjögren's Syndrome patients without visceral involvement, the most common treatments are moisture replacement therapies and immunosuppressants such as especially hydroxychloroquine (HCQ) and prednisone [8]. Treatment choice is based on the physicians' clinical experience, expert opinion, and limited clinical research [9, 10]. Chinese herbal medicine (CHM) is widely used in the treatment of pSS in China and improves patients' subjective symptoms in some clinical research [11, 12]. Sjögren's syndrome belongs to the “dryness arthralgia” category in traditional Chinese medicine. Following the pathogenesis of “deficiency, stasis, and toxicity,” we set up JieDuTongLuoShengJin, which is a recipe that has been felt to be effective in the bedside treatment of pSS. This study contributes to the ongoing research in effective, evidence-based interventions for Sjögren's syndrome.

## 2. Patients and Methods

### 2.1. Patient Selection

All pSS patients admitted to Yueyang Hospital of Integrated Traditional Chinese and Western Medicine affiliated with Shanghai University of Traditional Chinese Medicine between August 2014 and February 2016 were potential study candidates. All patients were between 18 and 70 years of age and fulfilled the American European Consensus Group criteria for pSS [13]. Eligibility criteria were ESSDAI score of <5, being without visceral involvement, and belonging to Yin deficiency on the basis of the traditional Chinese medicine. In addition, results from a salivary gland biopsy performed within 12 months before inclusion and showing the characteristic features of SS had to be available. Patients were naive to prior treatment with disease-modifying antirheumatic drugs (DMARDs), or if they had had previous treatment, then treatment of those treated with hydroxychloroquine or prednisone had to be discontinued at least 1 month before baseline, and treatment of those treated with other DMARDs had to be discontinued at least 6 months before baseline. Patients were permitted to use artificial tears and saliva, but the regimen had to remain identical during follow-up, and they had to be stopped at least 1 day prior to each assessment. Patients with a history of any malignancy or with underlying cardiac, cerebrovascular, pulmonary, liver, renal, or hematopoietic system conditions, chronic or latent infectious diseases (e.g., Human Immunodeficiency Virus and Acquired Immune Deficiency Syndrome (HIV/AIDS) and hepatitis C virus (HCV) infection), immune deficiency, and macular degeneration and pregnant women were excluded. During the study, patients who had been in other clinical trials were excluded. For patients who had emerging extraglandular manifestations, those who developed these within four weeks of study initiation were removed from the study; after four weeks they were terminated early from the study but their data kept for analysis. All patients underwent laboratory assessment, electrocardiography, and chest radiography at baseline.

### 2.2. Study Design

The study was conducted according to the Declaration of Helsinki. The protocol was reviewed and approved by the institutional review board and ethics committee of Yueyang Hospital of Integrated Traditional Chinese and Western Medicine affiliated with Shanghai University of Traditional Chinese Medicine. And it was registered in Chinese Clinical Trial Registry (registration ID: ChiCTR-IPR-14005441). All patients provided their written informed consent. According to literature review data, the combination of traditional Chinese and western medicine treatment efficacy rate is about 80% (*p*_1_), and that for pure western medicine treatment is 30% (*p*_2_), and *α* = 0.05 and *β* = 0.10. We formed the JieDuTongLuoShengJin granule group and placebo group in a ratio of 1 : 1. Using the formula *n* = (*z*_*α*_ + *z*_*β*_)^2^2*p*(1 − *p*)/(*p*_1_ − *p*_2_)^2^, where *n* is the number of each group, *p* = (*p*_1_ + *p*_2_)/2. We calculated that the required number of cases was 34. Taking into account the lost cases, 40 cases were designated for inclusion in this trial. 40 patients enrolled in this prospective, single-center, randomized, double-blind, placebo-controlled study were randomly divided into treatment group and control group (1 : 1) by SPSS 19.0 statistical software packages. The random coding table was stored in the clinical research database. A designated person was responsible for distributing the oral granules in accordance with the eligible patients' visiting sequence and test drug coding. All investigators and patients were blinded to treatment allocation. The dosage of placebo was a 1/10 dose of the JieDuTongLuoShengJin granules received by the treatment group. All patients in both treatment and placebo groups also received hydroxychloroquine (HCQ) 0.2 mg by mouth twice daily. Data for clinical efficacy and safety follow-up based on physical examination and laboratory tests were collected at week 12. In addition, AEs and serious AEs were evaluated. An adverse event (AE) is any adverse change in health or “side-effect” including any symptoms, syndromes, or diseases observed in a patient during the clinical trial.

### 2.3. Outcome Parameters 

#### 2.3.1. Definition of Endpoints

The primary endpoint was defined as a significant improvement in the ESSPRI score in the JieDuTongLuoShengJin granules group compared with the placebo group. Secondary endpoints were an improvement in the ESSDAI score and measurement of salivary and lacrimal gland function. Laboratory variables were also measured. All variables were assessed at baseline (within 2 weeks before treatment) and at 4 and 12 weeks after treatment.

#### 2.3.2. Evaluation of Disease Activity

The European League Against Rheumatism (EULAR) developed both consensus disease activity indexes used in this study: EULAR Sjögren's Syndrome Patient-Reported Index (ESSPRI) [14] and EULAR Sjögren's Syndrome Disease Activity Index (ESSDAI) [15].

The ESSPRI is a validated tool for subjective self-assessment of pSS disease activity. It contains three components: dryness, fatigue, and pain (joint and/or muscle pain), with a single 0–10 numerical scale for each of the three domains to assess patients' symptoms. The final score is obtained by averaging the scores of the three scales: (dryness + fatigue + pain)/3. The final ESSPRI score ranges from 0 to 10. The patient-acceptable symptom state (PASS) estimate has been defined as an ESSPRI < 5 points and the minimal clinically important improvement (MCII) as a decrease of at least one point or 15% [16].

The ESSDAI is a validated tool for global assessment of pSS disease activity by experienced clinicians. It includes 12 domains: constitutional, lymphadenopathy, glandular, articular, cutaneous, pulmonary, renal, muscular, peripheral nervous system, central nervous system, hematological, and biological domains. For each domain, features of disease activity are classified into three to four levels (0 = “no”; 1 = “low”; 2 = “moderate”; 3 = “high”) according to their severity. Each domain has a weight that ranges from 1 to 6. The final score is obtained by multiplying the disease domain by the domain weight and then summing the scores of all domains. ESSDAI ranges from 0 to 123. The MCII is defined as a decrease of at least three points. The EDDSAI is divided into three levels: low activity (ESSDAI < 5), moderate activity (5 ≤ ESSDAI < 14), and high activity (ESSDAI ≥ 14) [16].

The ESSPRI score was completed for each patient at baseline, at week 4, and at study completion. The ESSDAI scores were completed for each patient at baseline and at study completion. In addition, patients completed a Patient Global Assessment (PGA), which evaluates the global severity of their symptoms related to their pSS using a 0–10 numerical scale. All questionnaires were conducted through in-person interviews due to participants' low education level.

#### 2.3.3. Evaluation of Salivary and Lacrimal Gland Function

Unstimulated saliva can reflect salivary gland function. We collected unstimulated saliva using Lashley cups. Salivary volume was measured over 15 minutes between 1:00 and 4:00 PM on the day of assessment in order to minimize fluctuations related to the circadian rhythm of salivary secretion [17–19].

Three tests were used to evaluate lacrimal gland function: Schirmer's test I, breakup time (BUT), and the van Bijsterveld Ocular Dye Score [20, 21]. Schirmer's test I was carried out by placing a tear test filter paper in the lower fornix of the conjunctiva of the eye. The length of wetting was measured after 5 minutes. The BUT is the interval between the last blink and the appearance of the first randomly distributed dry spots. It is assessed by instilling a 1% fluorescein solution in the fornix of both eyes. The BUT was checked by the same physician for all patients. The van Bijsterveld Ocular Dye Score was calculated by the same ophthalmologist for all patients, too. The lissamine green test was performed by instilling 1% lissamine green in both eyes. After 1 or 2 full blinks, black dots appeared in the ocular surface, which were observated by cobalt blue with a slit lamp microscope. The intensity of staining of both cornea and bulbar conjunctiva was scored. The ocular surface staining score was rated from 0 to 9 (up to 3 points for each section [1 = sparsely scattered, 2 = densely scattered, and 3 = confluent]) [22, 23].

#### 2.3.4. Laboratory Assessment

Levels of immune globulin G (IgG), rheumatoid factor (RF), and C-reactive protein (CRP) were measured using rate nephelometry. The erythrocyte sedimentation rate (ESR) was measured with the Westergren method. Antinuclear antibodies (ANAs) were measured by indirect immunofluorescence and considered positive at a titer 1 : 80. Antibodies to SSA and SSB cellular antigens were determined using their enzyme linked immunosorbent assay (ELISA) kits.

### 2.4. Statistical Analysis

All statistical analyses involved use of SPSS 19.0 statistical software packages. The Shapiro-Wilk test was used to determine normality of continuous variables. Categorical data was summarized using counts/percentages. For continuous variables, those with normal distribution were analysed using the *t*-test and presented as “means ± standard deviation.” Those variables with abnormal distribution were analysed using the Mann–Whitney *U* test and presented as “median (interquartile range).” Categorical variables were analysed using the chi-square test or Fisher's exact probability test. Analysis of covariance (ANCOVA) and logistic regression analysis were used for analysis of postintervention group differences. In the intent-to-treat analyses, a last observation carried forward (LOCF) procedure was employed to impute missing clinical efficacy data (e.g., ESSPRI, ESSDAI, or PGA) at week 12. Only two-sided tests were applied. All efficacy analyses were based on the intention-to-treat principle, and, for all statistical analyses, a *p* value < 0.05 was considered statistically significant.

## 3. Results

### 3.1. Patient Characteristics

**Figure 1:**
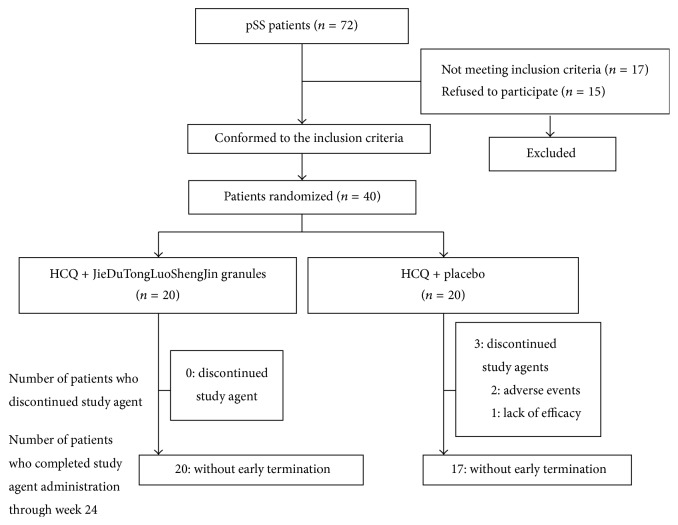
Patient disposition through week 12 of the trial. Randomization of patients with pSS into the two groups. Of a cohort of 183 patients, 40 patients were randomized into two treatment groups. HCQ: hydroxychloroquine.

40 patients were recruited and randomly assigned to this randomized, double-blind, placebo-controlled study (Figure 1). All patients were followed for 12 weeks, with the first exam performed on 26 August 2014 and the last week 12 evaluation completed on 15 May 2016.

Patients' baseline characteristics were generally comparable between the two groups (Table 1). We found no differences in the characteristics such as sex, age, and clinical features. 20 patients who took JieDuTongLuoShengJin granules orally were identified with an average age of 51.6 ± 11.28 years (versus 52.3 ± 12.40 years in the placebo group) and an average disease duration of 5.6 ± 5.12 years (versus 5.6 ± 3.65 years in the placebo group), and women constituted all of the population (versus 95.0% of the placebo group). There were no statistically significant differences in any pSS characteristics between the two groups (*p* > 0.05).

### 3.2. Clinical Efficacy

#### 3.2.1. Disease Activity

After treatment, the between-group and within-group before-and-after paired comparison results were analysed. The ESSPRI (the primary endpoint) (Tables 2 and 3) significantly improved in both groups (*p* = 0.009 at week 4 and *p* = 0.000 at week 12 in the treatment group, *p* = 0.039 at week 4, and *p* = 0.032 at week 12 in the placebo group, versus baseline), and a statistically significantly lower ESSPRI score was found in the JieDuTongLuoShengJin granules + HCQ group at week 12 (*p* = 0.002). Similar pattern of improvement was also observed for PGA (Table 3). The proportion of patients in the JieDuTongLuoShengJin granules + HCQ group achieving PASS increased from 45.00% at week 0 to 95.00% at week 12 (*p* = 0.001) and was significantly greater than that of the placebo + HCQ group (60.00%) (Table 2). The percentage of those in the JieDuTongLuoShengJin granules + HCQ group who achieved MCII (85.00%) was also significantly greater than that in the placebo + HCQ group (50.00%) at week 12 (Table 2).

Compared to week 0, assessments of each domain of ESSPRI also indicated significant improvement with JieDuTongLuoShengJin granules + HCQ at week 4 (*p* = 0.049, *p* = 0.042, and *p* = 0.034, resp.) and at week 12 (*p* = 0.011, *p* = 0.000, and *p* = 0.006, resp.). Patients in the placebo + HCQ group demonstrated significantly greater improvement in the pain domain (*p* = 0.025 at week 4 and *p* = 0.014 at week 12), but not dryness domain and fatigue domain. There was no statistical difference between the two groups in each domain of ESSPRI at week 4 (*p* > 0.05). At week 12, patients in the JieDuTongLuoShengJin granules + HCQ group demonstrated a statistically significant reduction in the dryness score and fatigue score versus placebo + HCQ (*p* = 0.026 and *p* = 0.002, resp.), but the differences for the pain score were not significant (*p* > 0.05) (Table 3).

The ESSDAI score was one of the secondary endpoints. All of the patients showed low disease activity without organ involvement at the time of week 0. The ESSDAI significantly improved in both groups at week 12 (*p* < 0.01 in the treatment group and *p* < 0.05 in the placebo group, versus baseline). But no significant difference was found between the two groups (*p* > 0.05) (Table 3), and none of the patients achieved MCII (Table 2).

#### 3.2.2. Salivary and Lacrimal Gland Function

At week 4, the unstimulated whole salivary flow rate increased to a certain extent in the treatment group, but there was no statistical difference (*p* > 0.05), and did not improve significantly in the control group (Table 3). The unstimulated whole salivary flow rate significantly increased from baseline in the treatment group (*p* = 0.000) more than in the placebo group (*p* = 0.049) at week 12; however, this difference was not significant between the two groups at week 12 (*p* > 0.05) (Table 3).

The between-group and within-group before-and-after paired comparison results of Schirmer's test, BUT test, and Corneal and Conjunctival staining were analysed and showed no significant changes in lacrimal gland function in either groups (*p* > 0.05) (Table 3).

#### 3.2.3. Laboratory Assessment

ESR level was significantly lower from baseline after treatment in both groups, but not for the levels of serum complement (C3 and C4) as well as CRP. Serum IgG lever improved significantly from baseline in patients treated with JieDuTongLuoShengJin granules (*p* = 0.010), but not in patients treated with placebo (*p* = 0.096). All laboratory variables mentioned above had no statistical differences between the two groups after treatment at week 12 (Table 3).

#### 3.2.4. Safety

During the study, adverse event happened in four patients. Similar proportions of patients from the treatment and placebo groups reported adverse event through week 4 (5.0% and 0.0%, resp.; *p* > 0.05) and week 12 (5.0% and 15.0%, resp.; *p* > 0.05) (Table 4). The most common adverse events were “erythra.” The one in the treatment group occurred at week 6, presenting as an itchy rash on the scalp that improved after cleaning the skin. Because it improved after skin cleaning, this was considered to be unrelated to the drug and the patient continued drug therapy and this patient was therefore not removed from the study. On the other hand, the adverse event of “erythra” in placebo group occurred at week 4, with a rash on the surface of limbs accompanied by itching. The symptom disappeared after stopping drug and appeared again when taking drugs. Therefore, the erythra that happened in this patient was considered to have likely been related to the use of hydroxychloroquine. There was one occurrence of pancreatitis, which was considered to be associated with poor primary disease control due to poor medication effectiveness, but the condition was not serious. Only one case of serious adverse events occurred in the placebo group. The patient was found to have abnormal liver function during follow-up at the time of week 4, which was confirmed as being due to hepatitis B virus (HBV) and therefore unrelated to this study. The three above patients in the placebo group with adverse events were excluded from this study.

## 4. Discussion

Primary Sjögren's syndrome is an incurable chronic disease with long disease duration and frequent flares and can cause multisystem damage in affected patients. At present, the treatment of Sjögren's syndrome is comprised of traditional immunosuppressants, and one of the most widely used immunosuppressants is hydroxychloroquine (HCQ).

Sjögren's syndrome belongs to the “dryness arthralgia” category in traditional Chinese medicine. Basic Questions first proposed the pathogenesis theory that Yin deficiency can transform into dryness and pointed out the treatment principle of nourishing Yin fluid. Body fluid and blood are derived from a common source, and severe consumption or loss of body fluid will affect the source of blood, leading to stasis of blood. The disease mechanism theory of “dryness-toxicity” was put forward by modern physicians of traditional Chinese medicine, referencing the “toxin factor theory” of epidemic febrile disease. According to this theory, patients suffering from these diseases share common characteristics of stubborn illness, long duration, severe symptomatology, and widespread physical involvement. These are also features of Sjögren's syndrome. Therefore, in traditional Chinese medicine, pSS is induced by “dryness-toxicity” and the blockade of qi and blood, which can lead to static blood and impairment of body fluid, and it results in Yin deficiency. Following these above principles, we set up JieDuTongLuoShengJin, which is a recipe that has been felt to be effective in the bedside treatment of pSS. It contains herbals that nourish Yin and promotes the secretion of fluid, relieves internal heat, promotes blood circulation, and removes obstruction in channels. We used radix paeoniae alba as sovereign drug to replenish qi and nourish blood; rhizoma zedoariae and angelica as minister drugs to strengthen the force of nourishing and invigorating blood circulation; spreading hedyotis herb as assistant drug to help relieve internal heat;* Astragalus membranaceus* as contrary drug to invigorate qi and promote circulation of blood; licorice root as an envoy drug to harmonize all of the above drugs. All the drugs were mixed together with the effect of detoxification, regulating the meridians, and promoting the secretion of saliva or body fluid.

Modern pharmacological evidence suggests that the total glycosides of paeoniae extracts from radix paeoniae alba could reduce serum immunoglobulin and erythrocyte sedimentation rate, improve symptoms such as xerophthalmia and xerostomia [24], and have bidirectional regulation effects on the proliferation of T and B lymphocytes to some extent, promoting or inhibiting the production of interleukin 1, interleukin 2, and tumor necrosis factor [25]. In previous experiments using nonobese diabetic mice, spreading hedyotis herb has been shown to improve body weight, water intake, salivary flow rate, and submandibular gland index [26]. Furthermore, it raises the expression of AQP5mRNA in salivary gland and submandibular gland tissue to some extent and this and additional medications may be able to increase the amount of AQP5, which can spur moisture transport rate, increase a cell's cytoplasmic volume, promote gland secretion, and therefore improve the symptoms of xerostomia [27, 28].

Previous research using JieDuTongLuoShengJin granules has demonstrated reduced immune globulin and the submandibular gland index in rat models of Sjögren's syndrome [29, 30]. The above evidence suggests that JieDuTongLuoShengJin is a very good immune regulator.

Our data in early clinical studies reveals that JieDuTongLuoShengJin use can improve some subjective symptoms and objective indicators in pSS [11]. This clinical trial aimed to verify those findings using a randomized, placebo-controlled, double-blind method. Patients with mild disease activity and without visceral involvement of pSS received oral JieDuTongLuoShengJin granules twice daily, or placebo granules, both in combination with a stable dose of HCQ.

In order to evaluate disease more comprehensively, we chose both the EULAR SS patient-reported index (ESSPRI) (the primary endpoint) and the EULAR SS disease activity index (ESSDAI) (the most important secondary endpoint) as the index of subjective symptoms and systemic features, two complementary ways to quantitatively measure disease severity that have been shown to be valid and sensitive measures [31, 32]. The ESSPRI score correlates well with other measures such as Patient Global Assessment (PGA), Profile of Fatigue and Discomfort (PROFAD) [33], and Sicca Symptoms Inventory (SSI) [34]. The ESSDAI score has better sensitivity [15] compared to other scales such as Sjögren's Syndrome Disease Activity Index (SSDAI) [35] and Sjögren's Systemic Clinical Activity Index (SCAI) [36]. There is no significant correlation between the two indices, but both are sensitive to changes in disease activity [37]. This study demonstrates that JieDuTongLuoShengJin, a traditional Chinese medicine, is overall superior to placebo in improving patients' subjective symptoms using the ESSPRI score (*p* < 0.01) and that each of the domains within the ESSPRI score decreased observably after treatment (*p* < 0.05). More than three-quarters of patients achieved MCII in the treatment group, significantly higher than the control group (*p* < 0.05). JieDuTongLuoShengJin granules + HCQ treatment also showed significant PGA improvement (*p* = 0.008 at week 4 and *p* = 0.000 at week 12), and although there was improvement in the ESSDAI score at week 12 (*p* = 0.003), it was not obviously different from that in the placebo group (*p* = 0.790). Therefore the combination of JieDuTongLuoShengJin granules with HCQ is superior to placebo combined with HCQ in improving subjective pSS symptoms, but not in modifying the degree of disease activity.

Furthermore, this study revealed that the levels of serum ESR diminished significantly in JieDuTongLuoShengJin + HCQ group. There was also an improvement in unstimulated saliva flow rate and reduced serum IgG, but this was not significantly different from that in the placebo group. Furthermore, neither treatment nor placebo groups showed CRP improvement. These results were not completely consistent with previous related studies, which have shown that HCQ can improve patient's fatigue and joint symptoms, and effectively reduce immunoglobulin, ESR, and CRP levels [38, 39]. In our present study, we chose the patients with low-activity-level pSS (ESSDAI < 5) but without visceral involvement, and our observation period was relatively brief. This may partly explain why our results were different from those of previous studies. In addition, in previous clinical studies, HCQ has been identified in patients with pSS as having a role of decreasing the level of B cell activating factor belonging to the TNF family (BAFF) in the serum and glands, reducing the concentration of cholinesterase, triggering the increasing of stimulated saliva flow rates and tear secretion, and finally improving sicca symptoms [40–43]. Animal experiments [44] indicate that HCQ can obviously downregulate the expression of interleukin 1 beta (IL-1*β*) and tumor necrosis factor alpha (TNF-*α*) in local submandibular gland of nonobese diabetic (NOD) mice on the levels of gene transcription and protein synthesis. It can reduce the content of IL-1*β* and TNF-*α* in peripheral blood serum, increase saliva flow rate, and reduce submandibular gland tissue damage on pathological examination.

This study demonstrates that although dryness symptoms and salivary flow rate improved with treatment, the effect on ocular dryness was not dramatic in either group. There were no significant differences of Schirmer's test, BUT, and ocular surface damage score before and after treatment in both groups, similar to previous research by Akpek et al. [45]. It is possible that these objective features vary modestly over time and therefore did not clearly exhibit a sufficient sensitivity to change [46–49]. For this reason, these measures have not been emphasized in recent trials [23, 50].

It is worth noting that similar proportions of (JieDuTongLuoShengJin granules + HCQ)-treated and (placebo + HCQ)-treated patients reported adverse event (AEs) during the study, and half of these were unlikely to have been related to the treatments those patients received: one had erythra that was felt to be unrelated to the study, and the other had hepatitis B virus infection. JieDuTongLuoShengJin granules are therefore safe in clinical use.

In conclusion, when combined with HCQ as a treatment, JieDuTongLuoShengJin granules show good effect in improving the subjective symptoms and some objective indicators of pSS. These results indicate that combination with JieDuTongLuoShengJin granules is promising for relieving symptoms and helpful to patients overall. It is a safe and effective treatment strategy for patients with pSS and deserves further clinical and experimental research.

## Figures and Tables

**Table 1:** Summary of demographics and characteristics at baseline of pSS patients.

	JieDuTongLuoShengJin granules + HCQ	Placebo + HCQ	*p* value
Patients randomized, *n*	20	20	
Age (years)			0.857
Mean ± SD	51.6 ± 11.28	52.3 ± 12.40	
Median	52	53	
Range	(27, 69)	(24, 68)	
Gender, *n* (%)			1.000
Male	0 (0.00)	1 (5.00)	
Female	20 (100.00)	19 (95.00)	
Disease duration (years)			0.989
Mean ± SD	5.6 ± 5.12	5.6 ± 3.65	
Median (IQR)	4.5 (2, 8.25)	5 (3, 7.25)	
Range	(0.2, 20)	(0.5, 15)	
Dryness (0–10)			0.907
Mean ± SD	5.7 ± 2.81	5.8 ± 2.55	
Median (IQR)	5.5 (3, 7.25)	5.5 (4.75, 8)	
Fatigue (0–10)			0.957
Mean ± SD	5.6 ± 3.22	5.7 ± 2.64	
Median (IQR)	5 (3.75, 8.25)	5 (4, 7.25)	
Pain (0–10)			0.678
Mean ± SD	2.7 ± 2.25	2.4 ± 2.50	
Median (IQR)	3 (0, 4)	2 (0, 4)	
ESSPRI (0–10)			0.939
Mean ± SD	4.7 ± 2.09	4.6 ± 1.99	
Median (IQR)	5 (2.7, 6.4)	4.3 (3.8, 5.9)	
PASS, *n* (%)	9 (45.00)	11 (55.00)	0.752
PGA (0–10)			0.624
Mean ± SD	5.4 ± 2.19	5.8 ± 2.29	
Median (IQR)	6 (3, 7)	6 (4, 7)	
ESSDAI (0–124)			0.655
Mean ± SD	2.6 ± 1.31	2.4 ± 1.31	
Median (IQR)	3 (1, 4)	2 (1, 4)	
Salivary flow rate (ml/15 min)			0.989
Mean ± SD	0.8 ± 0.70	0.7 ± 0.62	
Median (IQR)	0.8 (0.1, 1.0)	0.7 (0.2, 1.0)	
Schirmer's test (left) (mm/5 min)			0.224
Mean ± SD	4.5 ± 3.82	4.0 ± 4.66	
Median (IQR)	3.8 (2.0, 5.3)	2.0 (1.0, 4.5)	
Schirmer's test (right) (mm/5 min)			0.126
Mean ± SD	4.3 ± 3.01	3.2 ± 3.31	
Median (IQR)	4.0 (2.0, 6.3)	2.0 (1.0, 4.0)	
BUT (Left) (second)			0.140
Mean ± SD	3.6 ± 1.54	3.0 ± 1.69	
Median (IQR)	3.0 (3.0, 4.0)	2.5 (2.0, 4.0)	
BUT (Right) (second)			0.633
Mean ± SD	3.4 ± 1.57	3.2 ± 1.39	
Median (IQR)	3.0 (2.8, 3.3)	3.0 (2.0, 4.0)	
van Bijsterveld score (Left)			0.622
Mean ± SD	3.5 ± 1.88	3.2 ± 1.94	
Median (IQR)	4.0 (2.0, 5.0)	3.0 (2.0, 4.0)	
van Bijsterveld score (Right)			0.695
Mean ± SD	3.0 ± 1.69	3.2 ± 1.51	
Median (IQR)	3.0 (2.0, 4.0)	3.0 (2.8, 4.0)	
IgG (g/L)			0.596
Mean ± SD	18.3 ± 3.07	19.2 ± 6.91	
Median (IQR)	17.8 (16.4, 20.8)	17.3 (14.3, 20.5)	
C3 (g/L)			0.204
Mean ± SD	0.9 ± 0.20	0.8 ± 0.20	
Median (IQR)	0.9 (0.8, 1.0)	0.9 (0.8, 1.0)	
C4 (g/L)			0.071
Mean ± SD	0.2 ± 0.05	0.2 ± 0.05	
Median (IQR)	0.2 (0.2, 0.2)	0.2 (0.1, 0.2)	
ESR (mm/h)			0.871
Mean ± SD	35.9 ± 25.74	34.4 ± 31.83	
Median (IQR)	36.5 (14.3, 46.3)	23.5 (18.0, 40.3)	
CRP (mg/L)			0.756
Mean ± SD	3.4 ± 2.69	3.4 ± 2.81	
Median (IQR)	2.4 (2.1, 3.2)	2.6 (1.7, 4.2)	
RF positive, *n* (%)	9 (45.0)	7 (35.0)	0.748
Autoantibodies			
Anti-SSA	14 (70.0)	16 (80.0)	0.716
Anti-SSB	4 (20.0)	3 (15.0)	1.000

**Table 2:** Comparison of clinical curative effect in the two groups after treatment at week 12 (*n*  (%)).

	JieDuTongLuoShengJin granules + HCQ	Placebo + HCQ	*p* value
			(between-group)
PASS, *n* (%)			
ESSPRI	19 (95.00)	12 (60.00)	0.024
MCII, *n* (%)			
ESSPRI	17 (85.00)	10 (50.00)	0.024
ESSDAI	0 (0.00)	0 (0.00)	—

**Table 3:** Summary of clinical efficacy of pSS patients.

	JieDuTongLuoShengJin granules + HCQ		Placebo + HCQ		*p* value (between-group)	
	Week 4	Week 12	Week 4	Week 12	Week 4	Week 12
Dryness (0–10)					0.118	0.026
Mean ± SD	5.1 ± 2.17	3.6 ± 2.41	5.6 ± 2.28	5.2 ± 2.31		
Median (IQR)	5.0 (3.0, 7.0)	3.5 (1.8, 5.3)	6.0 (4.8, 7.3)	5.5 (4.5, 7.0)		
*p* value versus week 0	0.049	0.011	0.214	0.124		
Fatigue (0–10)					0.765	0.002
Mean ± SD	5.2 ± 2.71	3.3 ± 2.00	5.3 ± 2.15	4.9 ± 1.98		
Median (IQR)	5.0 (4.0, 7.3)	3.0 (2.0, 5.0)	5.0 (4.0, 7.0)	5.0 (4.0, 6.3)		
*p* value versus week 0	0.042	0.000	0.090	0.115		
Pain (0–10)					0.853	0.322
Mean ± SD	2.3 ± 1.98	1.5 ± 1.61	2.2 ± 2.43	1.7 ± 2.25		
Median (IQR)	2.5 (0.0, 3.3)	1.0 (0.0, 2.3)	1.5 (0.0, 4.0)	0.5 (0.0, 2.3)		
*p* value versus week 0	0.034	0.006	0.025	0.014		
ESSPRI (0–10)					0.217	0.002
Mean ± SD	4.2 ± 1.60	2.8 ± 1.18	4.4 ± 1.72	3.9 ± 1.63		
Median (IQR)	4.3 (2.7, 5.2)	2.7 (2, 3.7)	4.3 (3.7, 5.0)	3.9 (3, 4.8)		
*p* value versus week 0	0.009	0.000	0.039	0.032		
PGA (0–10)					0.716	0.019
Mean ± SD	5.0 ± 1.75	3.3 ± 1.68	5.4 ± 2.03	4.7 ± 2.01		
Median (IQR)	5.0 (3.0, 6.3)	3.0 (2.0, 5.0)	5.0 (4.0, 7.0)	5.0 (3.0, 6.0)		
*p* value versus week 0	0.008	0.000	0.042	0.016		
Salivary flow rate (ml/15 min)					0.613	0.321
Mean ± SD	0.8 ± 0.71	1.1 ± 0.81	0.7 ± 0.55	0.9 ± 0.57		
Median (IQR)	0.9 (0.2, 1.0)	1.0 (0.6, 1.5)	0.7 (0.3, 1.0)	0.9 (0.8, 1.0)		
*p* value versus week 0	0.085	0.000	0.354	0.049		
Schirmer's test (left) (mm/5 min)					0.445	0.527
Mean ± SD	4.7 ± 3.89	5.0 ± 4.44	3.9 ± 2.78	4.1 ± 2.44		
Median (IQR)	3.0 (2.0, 5.3)	4.0 (2.0, 5.3)	3.0 (2.0, 5.0)	3.3 (2.0, 6.0)		
*p* value versus week 0	0.457	0.284	0.938	0.585		
Schirmer's test (right) (mm/5 min)					0.543	0.759
Mean ± SD	4.5 ± 2.67	5.0 ± 5.15	3.6 ± 1.96	4.1 ± 2.34		
Median (IQR)	4.0 (3.0, 5.0)	4.0 (2.0, 6.0)	3.0 (2.0, 5.0)	4.0 (2.0, 6.0)		
*p* value versus week 0	0.561	0.948	0.202	0.114		
BUT (left) (second)					0.527	0.928
Mean ± SD	3.7 ± 1.87	3.9 ± 1.66	3.0 ± 1.43	3.6 ± 1.36		
Median (IQR)	3.5 (2.0, 4.3)	4.0 (2.8, 5.0)	2.5 (2.0, 3.3)	3.0 (2.8, 4.3)		
*p* value versus week 0	0.717	0.663	0.776	0.108		
BUT (right) (second)					0.474	0.873
Mean ± SD	3.6 ± 1.38	3.6 ± 1.39	3.2 ± 1.15	3.4 ± 1.50		
Median (IQR)	3.0 (3.0, 4.0)	3.5 (3.0, 4.3)	3.0 (2.0, 4.0)	3.5 (2.0, 5.0)		
*p* value versus week 0	0.490	0.479	0.763	0.299		
van Bijsterveld (left) (score)					0.542	0.588
Mean ± SD	3.2 ± 1.50	3.5 ± 1.93	3.5 ± 1.67	3.1 ± 1.80		
Median (IQR)	3.0 (2.0, 4.0)	3.5 (2.0, 5.0)	3.0 (2.8, 4.3)	3.0 (1.8, 4.3)		
*p* value versus week 0	0.460	0.877	0.720	0.769		
van Bijsterveld (right) (score)					0.800	0.631
Mean ± SD	3.4 ± 1.60	3.4 ± 1.90	3.5 ± 1.67	3.2 ± 1.76		
Median (IQR)	3.5 (2.8, 4.0)	3.5 (2.0, 4.3)	3.5 (2.8, 4.3)	3.0 (2.0, 4.3)		
*p* value versus week 0	0.482	0.456	0.550	0.919		
ESSDAI (0–124)						0.790
Mean ± SD		2.0 ± 1.10		1.8 ± 1.46		
Median (IQR)		2.0 (1.0, 3.0)		2.0 (1.0, 3.0)		
*p* value versus week 0		0.003		0.016		
IgG (g/L)						0.857
Mean ± SD		16.7 ± 2.39		17.3 ± 5.77		
Median (IQR)		17.3 (15.3, 18.9)		16.2 (14.7, 17.0)		
*p* value versus week 0		0.010		0.096		
C3 (g/L)						0.878
Mean ± SD		1.0 ± 0.16		0.9 ± 0.18		
Median (IQR)		1.0 (0.9, 1.0)		0.9 (0.8, 1.0)		
*p* value versus week 0		0.054		0.101		
C4 (g/L)						0.616
Mean ± SD		0.2 ± 0.05		0.2 ± 0.05		
Median (IQR)		0.2 (0.2, 0.3)		0.2 (0.2, 0.2)		
*p* value versus week 0		0.065		0.087		
ESR (mm/h)						0.371
Mean ± SD		24.4 ± 16.15		26.1 ± 20.93		
Median (IQR)		22.0 (14.0, 31.0)		21.5 (14.0, 30.5)		
*p* value versus week 0		0.001		0.049		
CRP (mg/L)						0.807
Mean ± SD		3.3 ± 1.97		4.4 ± 5.91		
Median (IQR)		2.0 (1.9, 4.3)		2.9 (1.8, 5.8)		
*p* value versus week 0		0.391		0.605		

**Table 4:** Analysis of adverse events observed in patients in the treatment groups (*n*  (%)).

Event	JieDuTongLuoShengJin granules + HCQ (%)	Placebo + HCQ (%)
Week 4		
Erythra	1 (5.0)	0 (0.0)
Week 12		
Liver dysfunction	0 (0.0)	1 (5.0)
Erythra	1 (5.0)	1 (5.0)
Pancreatitis	0 (0.0)	1 (5.0)
